# Smartphone-Assisted Colorimetric Detection of Glutathione and Glutathione Reductase Activity in Human Serum and Mouse Liver Using Hemin/G-Quadruplex DNAzyme

**DOI:** 10.3390/molecules26165016

**Published:** 2021-08-19

**Authors:** Yufen Lai, Mengyan Li, Xiaofei Liao, Li Zou

**Affiliations:** School of Pharmacy, Guangdong Pharmaceutical University, Guangzhou 510006, China; fly1797093770@163.com (Y.L.); limengyan133xx1539@163.com (M.L.); liaoxiaofei8513@163.com (X.L.)

**Keywords:** smartphone, colorimetric assay, glutathione, glutathione reductase, hemin/G-quadruplex DNAzyme

## Abstract

Abnormal levels of reduced glutathione (GSH) and glutathione reductase (GR) are usually related to a variety of diseases, so it is of great significance to determine the GSH concentration and GR activity. We herein develop a smartphone-assisted colorimetric biosensor for the detection of GSH and GR activity in human serum and mouse liver using hemin/G-quadruplex DNAzyme. Firstly, an obvious color change from colorless to green can be observed, owing to the high peroxidase-like activity of hemin/G-quadruplex DNAzyme toward 2,2′-azino-bis(3-ethylbenzothiozoline-6-sulfonic acid) (ABTS). With the addition of GSH or GR, the H_2_O_2_-mediated oxidation of ABTS catalyzed by hemin/G-quadruplex DNAzyme is significantly inhibited, resulting in remarkable color fading. Therefore, the detection of GSH and GR activity can be achieved by observing the color transition or measuring the absorbance at 420 nm. The detection limit was estimated to be as low as 0.1 μM and 10 μU/mL for GSH and GR, respectively. More interestingly, the RGB values of the sensing system can be identified by the smartphone application (APP, color collect), which makes it an ideal format for on-site determination and point-of-care testing (POCT). In addition, the proposed method shows excellent selectivity and acceptable applicability for the determination of GSH concentration and GR activity in human serum samples and mouse liver tissues, which might hold great application potential in clinical diagnosis and drug screening.

## 1. Introduction

As a major antioxidant, reduced glutathione (GSH) plays a key role in the protection of cells against various oxidative stresses, xenobiotic metabolism, intracellular signal transduction, and gene regulation [1,2]. Abnormal levels of GSH are linked to many diseases such as hepatic injury, Alzheimer’s disease, diabetes, human immunodeficiency virus (HIV) infection, and cancer [3,4]. Glutathione reductase (GR), an important enzyme involved in metabolic processes, can catalyze the reduction of oxidized glutathione (GSSG) to GSH in the presence of β-nicotinamide adenine dinucleotide 2′-phophate hydrate (NADPH) [5]. Intracellular glutathione is usually maintained in the reduced state through the GR-catalyzed glutathione redox cycle. GR is also known to play an essential role in response to biological oxidative stress and associated with some diseases and conditions including the genesis of anxiety [6,7]. In addition, it has been found that the level of hepatic GR activity was significantly decreased in liver injury [8]. Therefore, it is of great importance for the sensitive detection of GSH and GR activity in physiological media.

Up to now, various analytical techniques including colorimetry [9], fluorescence [10,11], photoluminescence [12], and electrochemistry [13] have been employed for the detection of GR activity. Although the classic spectrophotometric methods based on the absorbance changes of NADPH or DTNB are still widely used for the determination of GSH and GR activity, they are relatively insensitive and vulnerable [14,15]. Hence, substantial efforts are still needed for the development of a sensing strategy to meet the demand for sensitive and accurate detection of GSH and GR activity. As a simple and prevailing strategy, the colorimetric method has attracted extensive interest because of its advantages of convenient instrumentation, low cost, and allowing direct bare-eye detection [16], which is well suited for point-of-care diagnostics. Guanine (G)-rich DNA oligonucleotides can associate with hemin to form hemin/G-quadruplex DNAzyme with notable peroxidase-like catalytic activity, which can catalyze the H_2_O_2_-mediated oxidation of 3,3′,5,5′-tetramethylbenzidine sulfate (TMB) or 2,2′-azino-bis(3-ethylbenzothiozoline-6-sulfonic acid) (ABTS), which is accompanied by a color change [17,18]. Compared with traditional protein enzymes, hemin/G-quadruplex DNAzyme exhibits a number of advantages such as high thermal stability, ease of synthesis, and low cost, which makes it ideal candidates for the development of colorimetric biosensors [19,20,21,22].

GSH could be oxidated by H_2_O_2_, which inhibits the H_2_O_2_-mediated oxidation of ABTS catalyzed by hemin/G-quadruplex DNAzyme. Herein, we utilize hemin/G-quadruplex DNAzyme to develop a smartphone-assisted colorimetric biosensor for the detection of GSH and GR activity in human serum and mouse liver. It involves two major sections: (1) GR catalyzes the NADPH-dependent reduction of GSSG to GSH; (2) the generated GSH inhibits the H_2_O_2_-mediated oxidation of ABTS. Consequently, an obvious color transition from green to colorless can be visualized by the naked eye or measured by UV-vis spectroscopy. Moreover, we utilize a smartphone application (APP, such as color collect) to monitor the changes in RGB (red, blue, green) values when the green color of the sensing system changes to colorless upon the addition of GSH, which can be measured by using the back camera on the phone [23,24]. Following data processing, the RGB values are converted into the GSH concentration and GR activity. Therefore, the determination of GSH and GR activity can be achieved by recording the RGB values of the sensing system using a smartphone application or measuring the absorbance at 420 nm with a UV-vis spectrophotometer.

## 2. Results

### 2.1. Principle of the Colorimetric Sensing Strategy

**Scheme 1:**
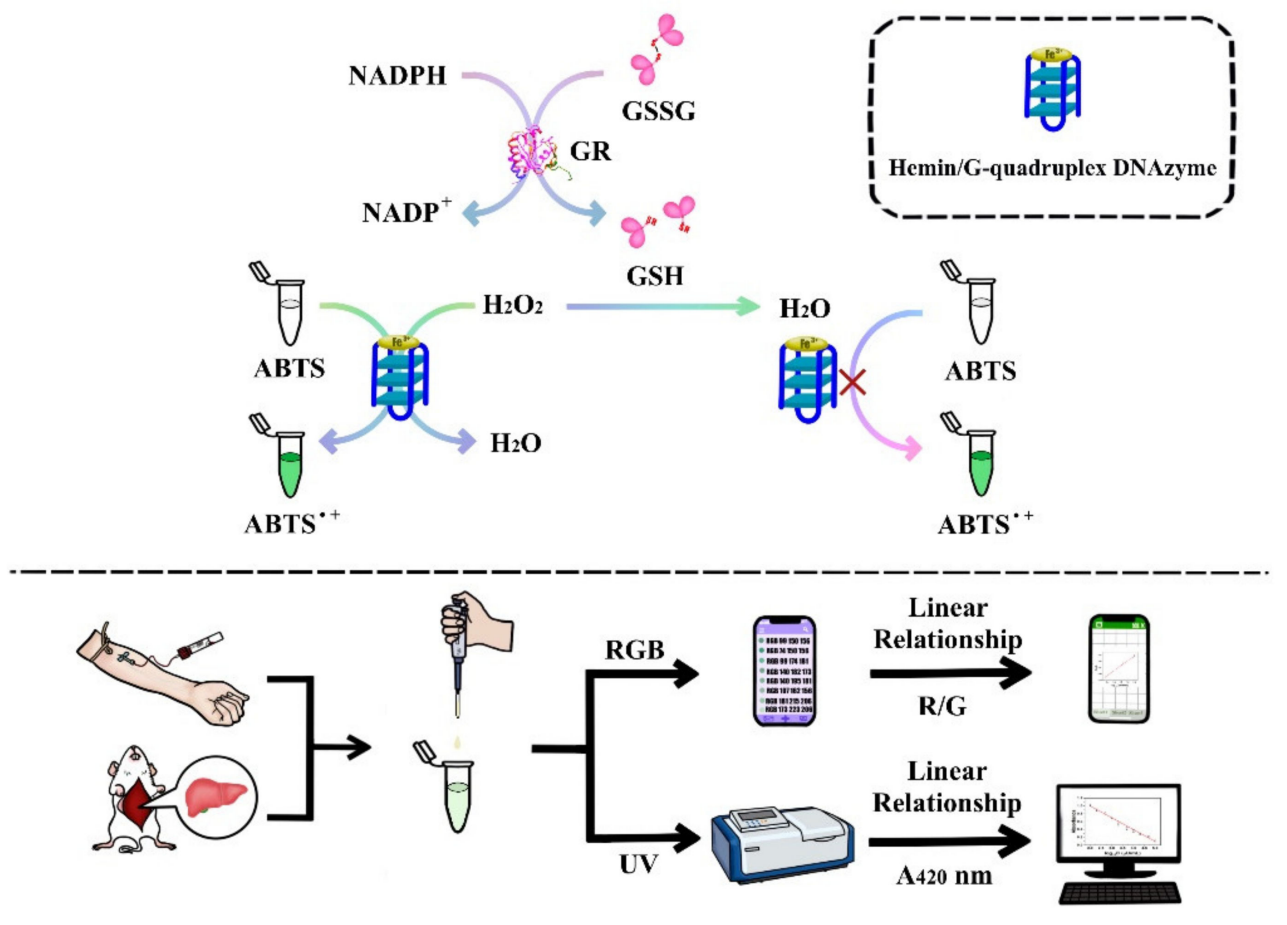
Schematic illustration of the colorimetric biosensor for the detection of GSH and GR activity in human serum and mouse liver using hemin/G-quadruplex DNAzyme.

The principle of the colorimetric detection of GSH and GR activity in human serum and mouse liver using hemin/G-quadruplex DNAzyme is illustrated in Scheme 1. Upon the addition of hemin into the sensing system, the G4-DNA folds into hemin/G-quadruplex DNAzyme, which can effectively catalyze the H_2_O_2_-mediated oxidation of ABTS to ABTS^·+^, accompanying an obvious color change from colorless to green. However, the addition of GSH to the sensing system significantly decreases the catalytic activity of hemin/G-quadruplex DNAzyme, resulting in a conspicuous color fading. Therefore, the colorimetric detection of GSH can be achieved by recording the color change using a smartphone or measuring the absorbance change at 420 nm using a UV-vis spectrophotometer. Considering that GR can catalyze the NADPH-dependent reduction of GSSG to GSH, the sensing strategy can be further applied to a colorimetric detection of GR activity.

### 2.2. Feasibility of the Colorimetric Sensing Strategy

**Figure 1:**
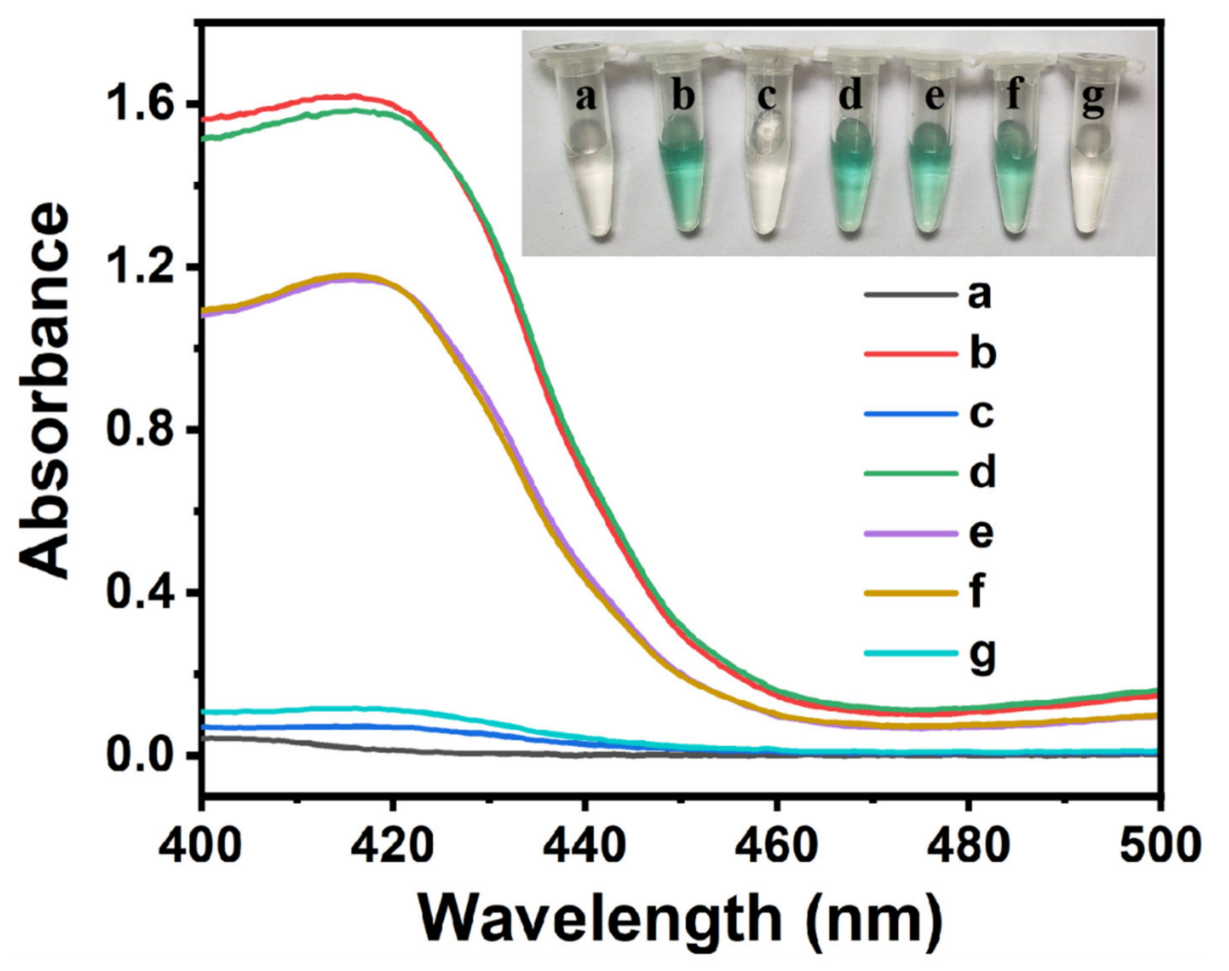
UV-vis absorption spectra of the sensing system under different conditions: (**a**) G4-DNA + hemin + ABTS, (**b**) a + H_2_O_2_, (**c**) b + GSH, (**d**) b + GSSG, (**e**) b + NADPH, (**f**) b + GSSG + NADPH, (**g**) f + GR. Inset: photographs.

To verify the feasibility of the hemin/G-quadruplex DNAzyme + ABTS-H_2_O_2_ sensing system for the colorimetric detection of GSH and GR activity, some control experiments were carried out. As shown in Figure 1, in the absence of H_2_O_2_, the solution was almost colorless, and no obvious UV-vis absorption peak was observed (curve a). When H_2_O_2_ was added into the system, the oxidation of ABTS occurred in the presence of hemin/G-quadruplex DNAzyme, which is evidenced by the green color and typical absorption peak at 420 nm (curve b). However, the color turned back to colorless upon the addition of GSH to the sensing system, and the absorbance at 420 nm was significantly decreased (curve c). These results indicated that the GSH showed a suppression effect toward the catalytic activity of hemin/G-quadruplex DNAzyme. In addition, the detection of GR activity was investigated by adding GSSG, NADPH, and GR into the sensing system. As expected, the addition of GSSG or NADPH or both of them could not induce an obvious color change of the sensing system, while NADPH itself showed an inhibition effect on the absorbance signal (curve e). In contrast, the coexistence of GSSG, NADPH, and GR could induce a remarkable color change from green to colorless, and significantly reduced absorbance was observed (curve g), owing to the generation of GSH via catalyzing the reduction of GSSG to GSH by NADPH-dependent GR. Therefore, the hemin/G-quadruplex DNAzyme + ABTS-H_2_O_2_ sensing system can be applied for the colorimetric detection of GSH and GR activity.

### 2.3. Optimization of the Experimental Conditions

**Figure 2:**
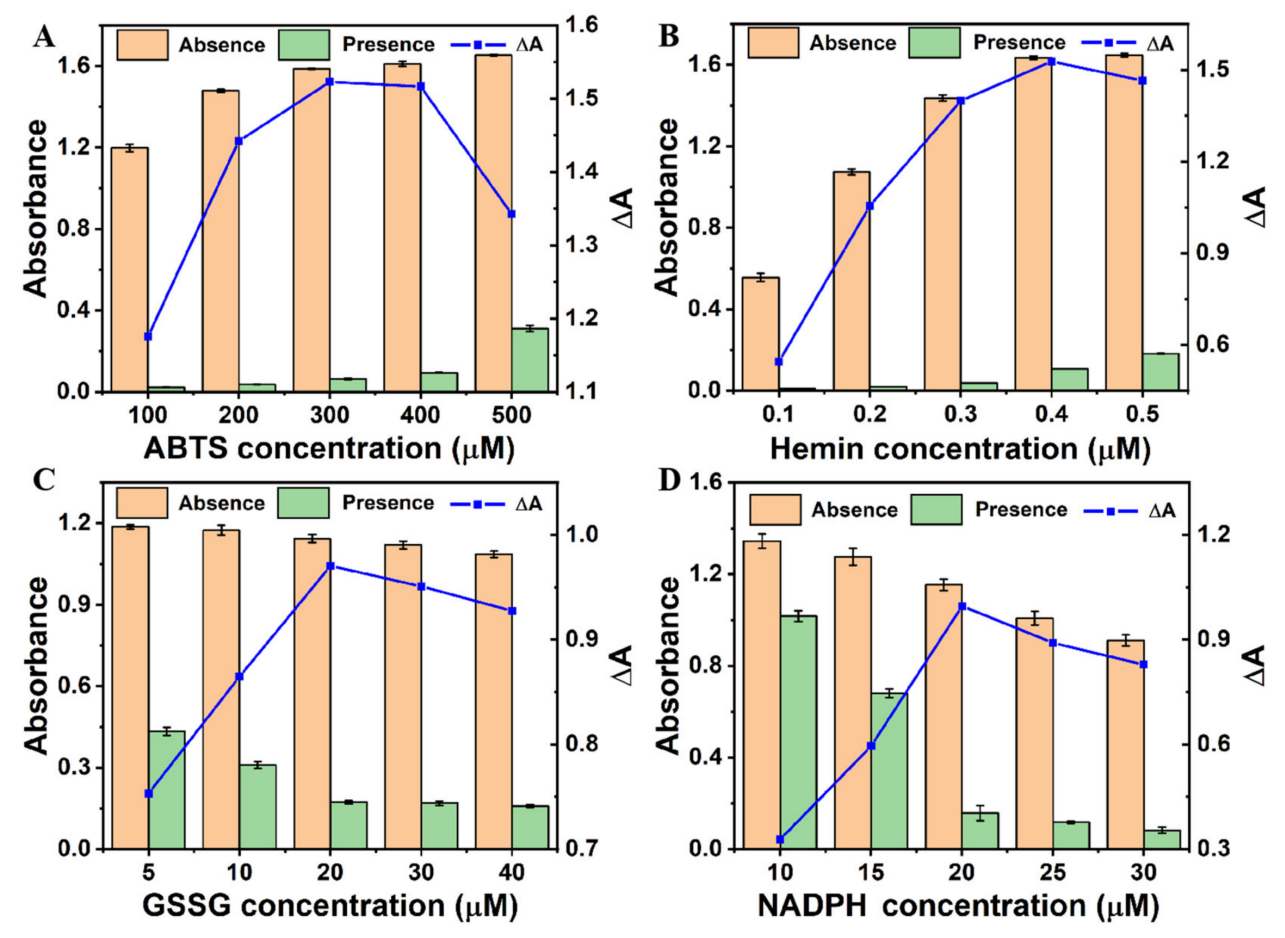
Optimization of the experimental condition: the concentrations of ABTS (**A**), hemin (**B**), GSSG (**C**), and NADPH (**D**). ΔA = A_0_ − A, where A_0_ and A were the absorbance at 420 nm of the sensing system in the absence and presence of GSH or GR, respectively. The error bars represent the standard deviation of three measurements.

The peroxidase-like activity of hemin/G-quadruplex DNAzyme is significantly influenced by the concentrations of ABTS and hemin. In order to achieve the best sensing performance, these experimental conditions were carefully optimized. Firstly, the influence of ABTS concentration was investigated. As shown in Figure 2A, the absorbance change (ΔA = A_0_ − A) increased significantly with the increase of ABTS concentration, and it reached the maximum value when the ABTS concentration was 300 µM (where A_0_ and A were the absorbance at 420 nm of the sensing system in the absence and presence of GSH, respectively). Therefore, 300 µM of ABTS was adopted in the following experiments. We further investigated the influence of hemin concentration. Figure 2B showed that the value of ΔA increased with the increasing hemin concentration from 0.1 to 0.4 µM, and it almost reached a plateau when the concentration was higher than 0.4 µM; thus, the hemin concentration of 0.4 µM was chosen as the optimal concentration for the subsequent experiments.

For GR activity detection, the concentrations of GSSG and NADPH were optimized, since they directly affect the amount of the generated GSH. It could be seen from Figure 2C that the value of ΔA increased significantly with the increase of GSSG concentration from 5 to 20 µM, which was followed by the decrease beyond 20 µM. As shown in Figure 2D, the ΔA reached the maximum value at 20 µM when changing the NADPH concentration from 10 to 30 µM. Therefore, 20 µM of GSSG and 20 µM of NADPH were chosen as the optimal conditions for further studies.

### 2.4. Analytical Performance

**Figure 3:**
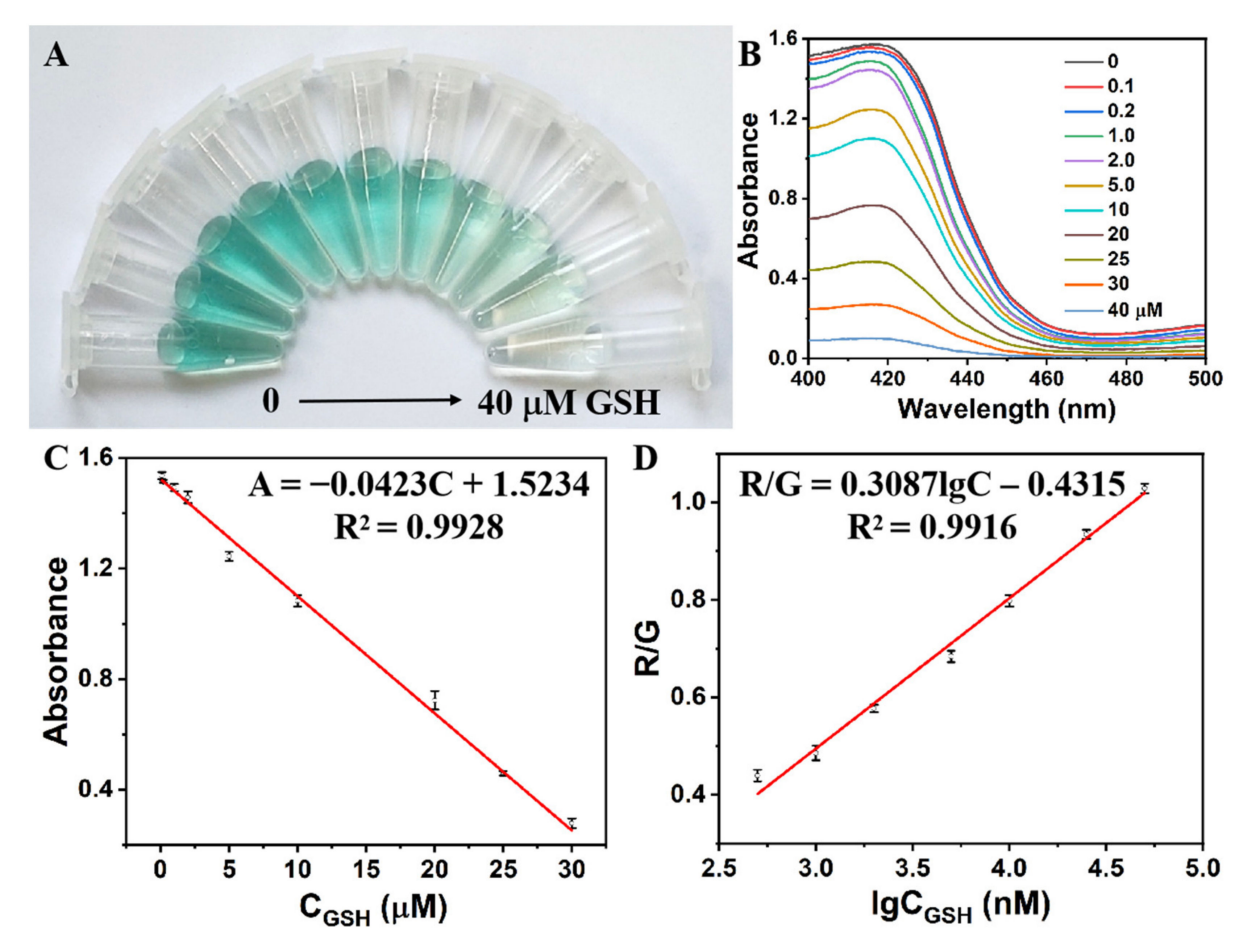
(**A**) Photographs showing colorimetric responses of the sensing system in the presence of various concentrations of GSH. (**B**) Their corresponding UV-vis absorption spectra. (**C**) Linear relationship between the absorbance at 420 nm and the concentration of GSH ranging from 0.1 µM to 30 µM. (**D**) The ratio of R/G versus the logarithmic value of GSH concentration in the range of 0.5–50 µM. The error bars indicate the standard deviation of three replicates.

Under the optimal experimental conditions, the analytical performance of the colorimetric sensing strategy for GSH detection was investigated. As shown in Figure 3A, the color of the solution gradually faded with the increase of the GSH concentration until 40 µM, where the solution changed to colorless. Accordingly, the largest absorption peak at 420 nm sharply decreased with increasing concentration of GSH from 0 to 40 µM (Figure 3B), after which it was nearly zero, indicating that the peroxidase-like activity of hemin/G-quadruplex DNAzyme has been totally inhibited. Notably, the absorbance at 420 nm displayed a linear relationship with the concentration of GSH in the range from 0.1 to 30 µM (Figure 3C). The limit of detection (LOD) was estimated to be as low as 0.1 µM with a signal-to-noise ratio of 3 [25], which was comparable to those of the previously reported methods for GSH detection (Table S1). Meanwhile, the RGB values of the sensing system processed with a smartphone were used to calculate the concentration of GSH. A linear relationship was found between the ratio of R/G [26] and the logarithmic value of GSH concentration in the range of 0.5–50 µM (Figure 3D), and a detection limit down to 0.2 µM was obtained. However, this method is cost-effective and more suitable for on-site determination and POCT. Several potential interferences were added into the sensing system to investigate the selectivity of our method for GSH detection. As displayed in Figure S1, the absorbance at 420 nm decreased significantly upon the addition of GSH, while these interfering substances exhibited no obvious absorbance changes, which indicates that the proposed sensing strategy exhibited excellent selectivity for GSH detection.

**Figure 4:**
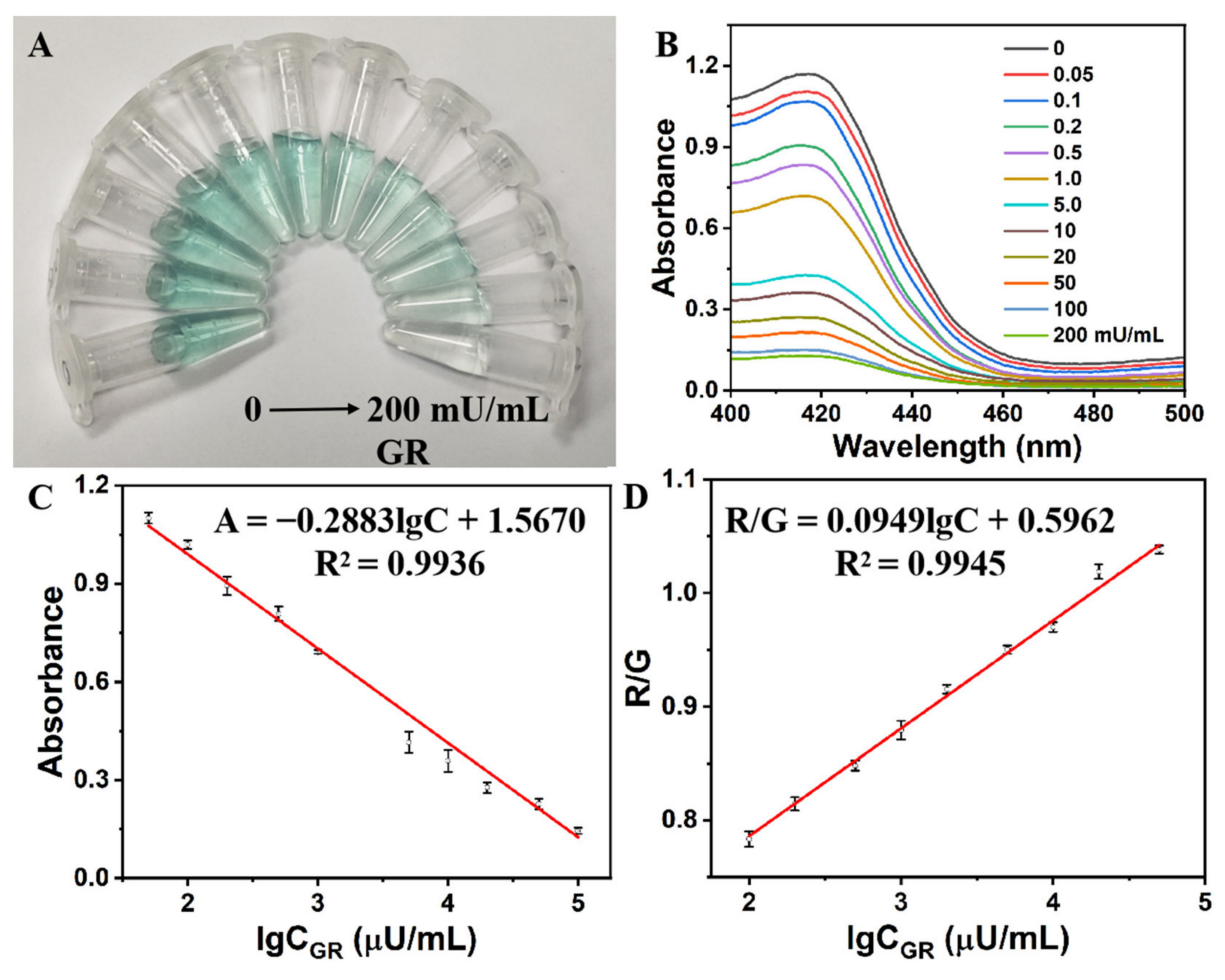
(**A**) Photographs and (**B**) UV-vis absorption spectra of the sensing system as a function of the concentration of GR. (**C**) Plot of the absorption intensity of the sensing system at 420 nm versus the logarithmic value of the concentration of GR. (**D**) Linear relationship of the ratio of R/G versus the logarithmic value of the concentration of GR. The error bars indicate the standard deviations of three measurements.

In addition, we evaluated the quantitative analysis capacity of the colorimetric sensing system for GR activity detection. Figure 4A displayed the color of the solution in the presence of different concentrations of GR; remarkable color fading was observed as the GR concentrations increased from 0 to 200 mU/mL. The corresponding absorption signal decreased gradually along with the increase of GR concentration (Figure 4B). Figure 4C presented the linear relationship between the absorbance at 420 nm and the logarithmic value of the concentration of GR ranging from 0.05 to 100 mU/mL. The detection limit of the proposed method for GR was estimated to be 10 µU/mL, which was comparable and superior to those of other GR detection methods (Table S2). Meanwhile, the colorimetric sensing system allowed the determination of GR down to 25 µU/mL by the RGB method, with a linear range from 0.1 to 50 mU/mL (Figure 4D). To investigate the selectivity of the sensing system for GR detection, several common proteins/enzymes were tested. As shown in Figure S2, no significant absorbance change was observed from these interferences, indicating that this assay was highly selective for GR, which is ascribed to the specific catalysis of GR toward GSSG.

### 2.5. Real Samples Assay

To evaluate the practical applicability, this method was applied for determination of GSH and GR in human serum using a standard addition method. Table 1 showed the analytical results of GSH concentration and GR activity in the spiked serum samples. According to the obtained calibration curves, the concentrations of GSH and GR in human serum sample were calculated to be 0.34 mM and 25.5 mU/mL, respectively, and the results were consistent with the previous reports [27,28]. Moreover, the recoveries were measured between 98.77% and 108.86% with the relative standard deviation (RSD) values lower than 9.27%, indicating that the colorimetric sensing system showed good anti-interference capacity for the detection of GSH and GR activity in human serum samples.

**Figure 5:**
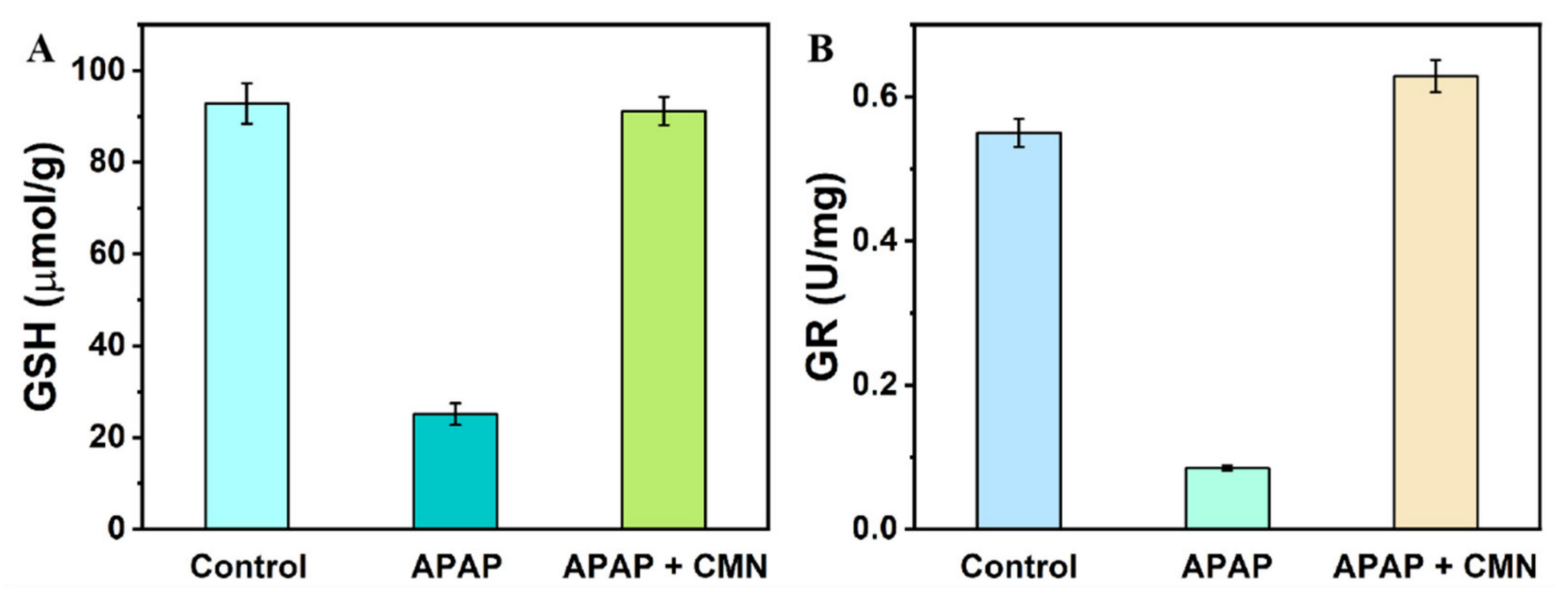
CMN pretreatment enhances the levels of hepatic GSH as well as GR activity after APAP. Liver homogenates were prepared to analyze the GSH level (**A**) and GR activity (**B**) 16 h after APAP injection. Data are expressed as mean ± SE; *n* = 3 mice per group.

We further applied the proposed method to assay the GSH concentration and GR activity in mouse liver tissues. As shown in Table 2, satisfactory results with recoveries in the range of 96.16–105.28% along with the RSD values lower than 9.36% were obtained, implying the validity of the proposed method for the detection of GSH and GR activity in mouse liver tissues. Moreover, this assay was used to explore the protective effect of CMN on acute liver injury induced by APAP in mice. Figure 5 showed the levels of GSH and GR activity in the liver of three experimental groups. APAP treatment significantly decreased in the levels of hepatic GSH as well as GR activity as compared to the control group. As expected, CMN pretreatment significantly showed recovery of the hepatic GSH content and GR activity. The protective effect was further confirmed by the hepatic histological findings (Figure S3); marked disruption of the hepatocytes structure, necrosis of liver cells, and inflammatory cell infiltration were observed in the mice treated with APAP. However, these histological changes were significantly suppressed by CMN pretreatment. Our study revealed that CMN increased the levels of antioxidants GSH and GR, thus reducing oxidative stress in the liver of APAP-treated mice.

## 3. Materials and Methods

### 3.1. Materials

Glutathione reductase (GR) from baker’s yeast, urease, glucose oxidase (GOx), trypsin, and acetaminophen (APAP) were purchased from Sigma-Aldrich (Shanghai, China). Reduced glutathione (GSH), oxidized glutathione (GSSG), hemin, bovine serum albumin (BSA), and curcumin (CMN) were obtained from Aladdin Chemical Reagent Co., Ltd. (Shanghai, China). Urea, folic acid (FA), glucose, arginine, 2,2′-azino-bis(3-ethylbenzthiazoline-6-sulfonic acid) (ABTS), and β-nicotinamide adenine dinucleotide 2′-phosphate reduced tetrasodium salt hydrate (NADPH) were purchased from Sangon Biotech Co., Ltd. (Shanghai, China). The stock solution of 5.0 mM hemin was prepared in dimethyl sulfoxide (DMSO) and stored in darkness at −20 °C. All other reagents were of analytical grade and used as received without further purification. Ultrapure water with a resistivity of 18.2 MΩ cm obtained from a Milli-Q purification system was used throughout the experiments. Oligonucleotide (5′-TTT GGG TAG GGC GGG TTG GG-3′, G4-DNA) was synthesized by Sangon Biotech Co., Ltd. (Shanghai, China).

### 3.2. Optimization of Experimental Conditions

To achieve the best analytical performance, some key experimental parameters were optimized, including various concentrations of ABTS (100–500 µM), hemin (0.1–0.5 µM), GSSG (5–40 µM), and NADPH (10–30 µM).

### 3.3. Colorimetric Detection of GSH and GR

In a typical experiment, the G4-DNA (2 µM) and hemin (0.4 µM) were mixed in a Tris-HAc buffer solution (20 mM, pH 7.5) and incubated at 37 °C for 30 min to form hemin/G-quadruplex DNAzyme. The GSH detection assay was conducted by adding ABTS (300 µM), H_2_O_2_ (6 mM), and GSH with different concentrations (0, 0.1, 0.2, 1.0, 2.0, 5.0, 10, 20, 25, 30, and 40 µM) to the as-prepared hemin/G-quadruplex DNAzyme with further incubation for 6 min at room temperature. For GR activity detection, different concentrations of GR (0, 0.05, 0.1, 0.2, 0.5, 1.0, 5.0, 10, 20, 50, 100, and 200 mU/mL) were added to the Tris-HAc buffer containing 20 µM GSSG and 20 µM NADPH. The mixtures were incubated at 37 °C for 10 min to catalyze the reduction of GSSG to GSH. Subsequently, the GR-catalyzed product was added to the hemin/G-quadruplex DNAzyme + ABTS-H_2_O_2_ system; all the other conditions are the same with the GSH detection procedure mentioned above. Finally, the color of the resulting solution was recorded using the built-in camera of the smartphone, and the app color collect was adopted to read the RGB values from the photographs. In addition, the corresponding UV-vis absorbance spectra were measured with a UV-6100 spectrophotometer (Metash, Shanghai, China) in the wavelength range of 400–500 nm.

### 3.4. Selectivity of the Proposed Method

In order to investigate the selectivity of the proposed method, we added several potential interferences, including glucose, arginine, FA, and urea (40 µM) for GSH detection, BSA, urease, GOx, and trypsin (5 µg/mL) for GR detection, to the sensing system [10], in which the experimental procedures were almost the same as the GSH and GR activity assay.

### 3.5. Determination of GSH and GR in Human Serum and Mouse Liver

Human serum were kindly donated by Sun Yat-sen University Cancer Center (Guangzhou, China). The human serum samples from healthy volunteers were diluted 150 times with Tris-HAc buffer (20 mM, pH 7.5) and used for replacing the standard solutions in the above homogeneous assays. Then, different concentrations of GSH (0, 0.5, 2, and 25 µM) or GR (0, 0.05, 0.2, and 2 mU/mL) were added into the diluted serum samples to conduct the recovery experiments.

C57BL/6J mice (14–20 wk of age) were obtained from the Guangdong Medical Laboratory Animal Center (Foshan, China). The mice were raised in a stable environment at 22 °C, relative humidity 50% ± 5%, and a 12 h light/dark cycle. Approval for all animal experiments was obtained from the Institutional Animal Care and Use Committee at the Guangdong Pharmaceutical University. Mice were randomly assigned into three groups: control, APAP, and APAP + CMN group. The control group received the equivalent volumes of 0.9% sodium chloride (NaCl) intraperitoneally (ip); the APAP group received 0.9% NaCl 2 h (ip) before APAP injection; and the APAP + CMN group received CMN (20 mg/kg, ip) 2 h before APAP challenge. APAP was dissolved in 0.9% NaCl and injected at a single dose of 500 mg/kg. Mice were sacrificed at 16 h after APAP injection; immediately, livers were removed and washed with 0.9% NaCl. A part of liver was fixed in 10% formalin and embedded in paraffin for histological examinations. The tissue homogenates were prepared in ice-cold PBS using a bead mill homogenizer and then centrifuged at 8000× *g* for 10 min at 4 °C to collect supernatants for the quantitative determination of GSH level and GR activity. The liver samples were diluted 600 times for GSH detection and 150 times for GR detection.

## 4. Conclusions

In summary, we have developed a smartphone-assisted colorimetric biosensor for the sensitive detection of GSH and GR activity in human serum and mouse liver based on the peroxidase-like activity of hemin/G-quadruplex DNAzyme. The H_2_O_2_-mediated oxidation of ABTS can be catalyzed by hemin/G-quadruplex DNAzyme while being inhibited by the product GSH of a GR-catalyzed glutathione redox cycle. An obvious color transition from green to colorless can be visualized after the addition of GSH immediately, and the colorimetric readout can be recorded by a smartphone application or measured by UV-vis spectroscopy. The proposed method exhibits high sensitivity and selectivity for the determination of GSH and GR activity in real samples. Moreover, this assay can be applied to explore the protective effect of CMN on hepatic injury induced by APAP, which will likely open a new direction for the screening of active ingredients from traditional Chinese medicine using hemin/G-quadruplex DNAzyme-based bioassays.

## Figures and Tables

**Table 1:** Detection of GSH and GR in human serum sample ^1^ (mean ± SD ^2^, *n* = 3).

Analyte	Added	Found	Recovery (%)	RSD (%)
GSH (µM)	0	2.26 ± 0.13	–	5.93
	0.5	2.99 ± 0.07	108.54	2.22
	2	4.39 ± 0.34	103.12	7.66
	25	26.92 ± 0.39	98.77	1.45
GR (mU/mL)	0	0.17 ± 0.01	–	8.05
	0.05	0.24 ± 0.02	108.86	9.27
	0.2	0.40 ± 0.04	108.46	8.79
	2	2.26 ± 0.13	104.02	5.90

^1^ The serum samples were diluted 150-fold to ensure that the concentrations were in the detection range of the proposed method. ^2^ The standard deviation (SD) of three measurements.

**Table 2:** Detection of GSH and GR in mouse liver tissues (mean ± SD ^1^, *n* = 3).

Analyte	Added	Found	Recovery (%)	RSD (%)
GSH ^2^ (nmol/g)	0	89.87 ± 8.41	–	9.36
	30	117.88 ± 0.96	98.34	0.82
	120	220.95 ± 15.71	105.28	7.11
	600	663.37 ± 48.04	96.16	7.24
GR ^3^ (U/g)	0	3.48 ± 0.25	–	7.29
	0.6	4.12 ± 0.22	100.98	5.42
	3	6.67 ± 0.52	102.94	7.81
	15	19.08 ± 1.35	103.21	7.07

^1^ The standard deviation (SD) of three measurements. The liver samples were diluted 600-fold ^2^ and 150-fold ^3^ to ensure that the concentrations were in the detection range of the proposed method.

## Data Availability

Not applicable.

## Supplementary Materials

The following are available online, Figure S1: The absorbance at 420 nm of the sensing system in the presence of GSH and other potential interferences, Figure S2: The absorbance at 420 nm of the sensing system in the presence of GR and other proteins/enzymes, Figure S3: Histological changes of liver sections in different groups, Table S1: Comparison of the proposed sensing strategy with other GSH detection methods, Table S2: Comparison of the proposed sensing strategy with other GR detection methods.

## Sample Availability

Samples are not available from the authors.
