# Supplementary material for: Smartphone-Assisted Colorimetric Detection of Glutathione and Glutathione Reductase Activity in Human Serum and Mouse Liver Using Hemin/G-Quadruplex DNAzyme
Source: Molecules. 2021 Aug 19;26(16):5016. doi: 10.3390/molecules26165016 (PMC8398021; doi:10.3390/molecules26165016)
Supplement: Supplementary file 1 [file molecules-26-05016-s001.zip › molecules-1335096-supplementary.pdf]

Supplementary Material (SM)

Smartphone-assisted colorimetric detection of glutathione and glutathione reductase activity in human serum and mouse liver using hemin/G-quadruplex DNAzyme

Yufen Lai, Mengyan Li, Xiaofei Liao, Li Zou*

School of Pharmacy, Guangdong Pharmaceutical University, Guangzhou 510006, PR China

*Corresponding author.

E-mail: lizou@gdpu.edu.cn

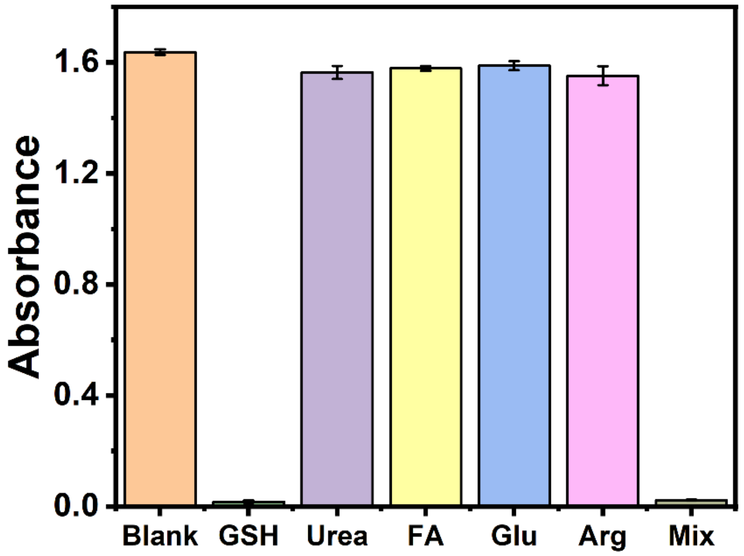

Figure S1. The absorbance at 420 nm of the sensing system in the presence of GSH and other potential interferences, including urea, folic acid (FA), glucose (Glu) and arginine (Arg). The error bars represent the standard deviations of three measurements.

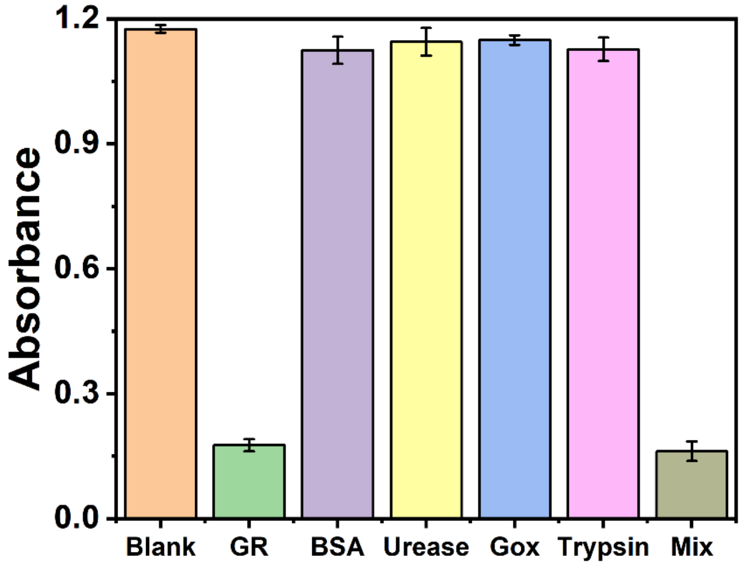

Figure S2. The absorbance at 420 nm of the sensing system in the presence of GR and other proteins/enzymes. The error bars indicate the standard deviations of three measurements.

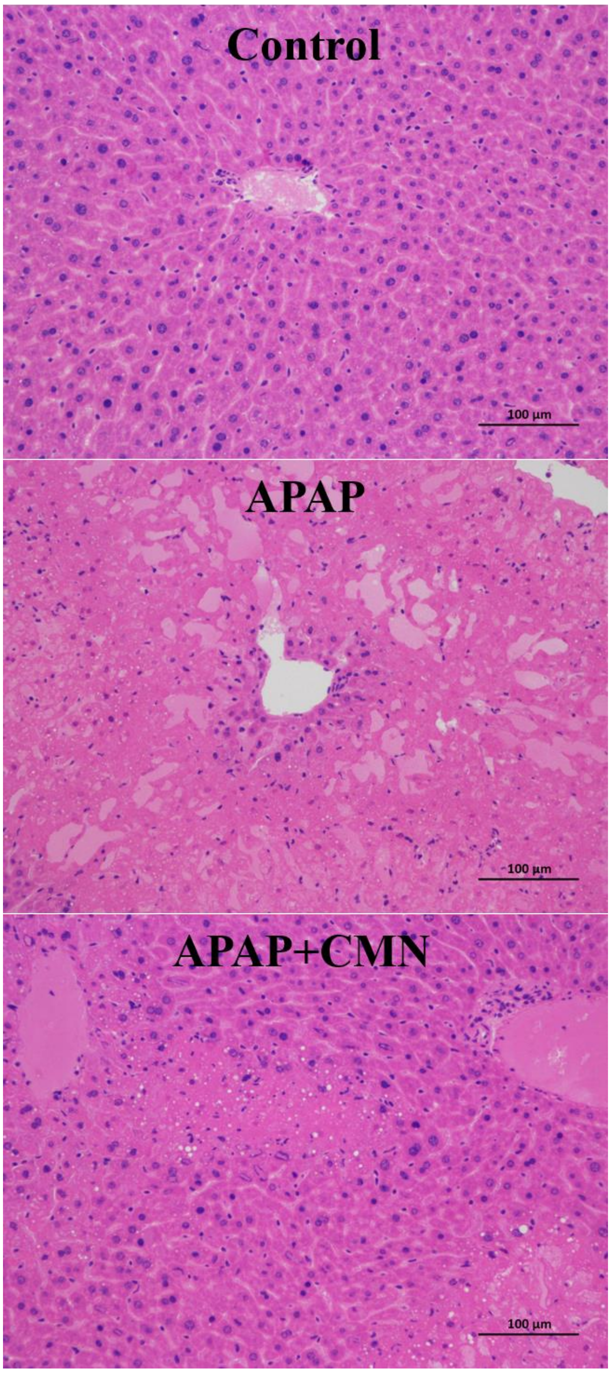

Figure S3. Histological changes of liver sections in different groups (HE stain ×200).

Table S1. Comparison of the proposed sensing strategy with other GSH detection methods.

Detection methods	Linear range (μM)	LOD (μM)	Analysis time (min)	References
Fluorescence	0.1–20	0.05	30	1
Fluorescence	0–150	2.86	60	2
Luminescence	1–200	0.13	120	3
Fluorescence	1–10	0.3	3	4
Fluorescence	5–250	0.6	–	5
Fluorescence	0–10	0.434	90	6
Fluorescence	2–104	0.9	–	7
Fluorescence	0.5–6	0.38	30	8
Colorimetry	0.1–30	0.1	36	This work

Table S2. Comparison of the proposed sensing strategy with other GR detection methods.

Detection methods	Linear range (mU/mL)	LOD (mU/mL)	Analysis time (min)	References
Fluorescence	0.02–30	0.01	60	1
Fluorescence	0.05–2	–	1080	9
Fluorescence	0.2–2	0.2	1	10
Fluorescence	4–40	0.5	10	11
Fluorescence	0.1–2	0.05	18	12
Electrochemistry	5–100	5	12	13
Photoluminescence	0.34–17	0.34	–	14
Fluorescence	0.005–0.13	0.005	20	15
Colorimetry	0.05–100	0.01	36	This work

References

1. Kong, R.; Ma, L.; Han, X.; Ma, C.; Qu, F.; Xia, L. Hg^{2+}-mediated stabilization of G-triplex based molecular beacon for label-free fluorescence detection of Hg^{2+}, reduced glutathione, and glutathione reductase activity. *Spectrochim. Acta A* **2020**, *228*, 117855.
2. Zhao, H.; Wen, X.; Li, W.; Li, Y.; Yin, C. A copper-mediated On-Off-On gold nanocluster for endogenous GSH sensing to drive cancer cell recognition. *J. Mater. Chem. B* **2019**, *7*, 2169–2176.
3. Dong, Z.; Lu, L.; Ko, C.N.; Yang, C.; Li, S.; Lee, M.Y.; Leung, C.H.; Ma, D. A MnO_2 nanosheet-assisted GSH detection platform using an iridium(Ⅲ) complex as a switch-on luminescent probe. *Nanoscale* **2017**, *9*, 4677–4682.
4. Cai, Q.; Li, J.; Ge, J.; Zhang, L.; Hu, Y.; Li, Z.; Qu, L. A rapid fluorescence "switch-on" assay for glutathione detection by using carbon dots-MnO_2 nanocomposites. *Biosens. Bioelectron.* **2015**, 72, 31–36.
5. Liu, J.; Bao, C.; Zhong, X.; Zhao, C.; Zhu, L. Highly selective detection of glutathione using a quantum-dot-based OFF–ON fluorescent probe. *Chem. Commun.* **2010**, *46*, 2971–2973.
6. Cui, M.; Li, W.; Wang, L.; Gong, L.; Tang, H.; Cao, D. Twisted intramolecular charge transfer plus aggregation-enhanced emission active based quinoxaline luminogen: photophysical properties and a light-up fluorescent probe for glutathione. *J. Mater. Chem. C* **2019**, 7, 3779–3786.
7. Amouzegar, Z.; Afkhami, A.; Madrakian, T. ZnS quantum dots surface-loaded with zinc (Ⅱ) ions as a viable fluorescent probe for glutathione. *Microchim. Acta* **2019**, *186*, 205.
8. Zhang, N.; Qu, F.; Luo, H.; Li, N. Sensitive and selective detection of biothiols based on target-induced agglomeration of silver nanoclusters. *Biosens. Bioelectron.* **2013**, *42*, 214–218.
9. Piggott, A.M.; Karuso, P. Fluorometric assay for the determination of glutathione reductase activity. *Anal. Chem.* **2007**, *79*, 8769–8773.
10. Zhu, S.; Zhao, X.; Zhang, W.; Liu, Z.; Qi, W.; Anjum, S.; Xu, G. Fluorescence detection of glutathione reductase activity based on deoxyribonucleic acid-templated silver nanoclusters. *Anal. Chim. Acta* **2013**, *786*, 111–115.
11. Li, Q.; Zhang, L.; Bai, J.; Liu, Z.; Liang, R.; Qiu, J. Preparation of novel fluorescent DNA bio-dots and their application for biothiols and glutathione reductase activity detection. *Biosens. Bioelectron.* **2015**, *74*, 886–894.
12. Zhu, S.; He, L.; Zhang, F.; Li, M.; Jiao, S.; Li, Y.; Chen, M.; Zhao, X.; Wang, H. Fluorimetric evaluation of glutathione reductase activity and its inhibitors using carbon quantum dots. *Talanta* **2016**, *161*, 769–774.
13. Wu, Y.; Jiang, L.; Ning, G.; Chu, L.; Liu, W.; Wang, Y.; Zhao, Y. A sensitive and simple impedance sensing strategy for glutathione and glutathione reductase activity detection. *J. Anal. Chem.* **2019**, *74*, 505–512.
14. Jiang, H.; Su, X.; Zhang, Y.; Zhou, J.; Fang, D.; Wang, X. Unexpected thiols triggering photoluminescent enhancement of cytidine stabilized Au nanoclusters for sensitive assays of glutathione reductase and its inhibitors screening. *Anal. Chem.* **2016**, *88*, 4766–4771.
15. Yan, X.; Zhao, X.; Sun, J.; Zhu, S.; Lei, C.; Li, R.; Gong, P.; Ling, B.; Wang, R.; Wang, H. Probing glutathione reductase activity with graphene quantum dots and gold nanoparticles system. *Sensors Actuators B Chem.* **2018**, *263*, 27–35.
